# A novel TYR variant implicated in oculocutaneous albinism type 1 (OCA1): a study in an indigenous population in Southern Brazil

**DOI:** 10.1007/s12687-026-00948-x

**Published:** 2026-09-24

**Authors:** Paulyana Corecco-Moura, Laércio Moreira Cardoso-Júnior, Osvaldo Alfonso Pinto Artigalás, Fernanda Sales Luiz Vianna, Ana Paula Pedroso Junges, Renan Rangel Bonamigo, Carmem Rafael Sales, Guilherme Ladwig Tejada, Patricia Ioschpe Gus, Luana da Silva Kaingang, Leocir Muller Ribeiro, Daniel Fernando Campos Sales, Daniela Sales Kaingang, Ana Paula Ornaghi, Marcia Holsbach Beltrame, Lavínia Schüler-Faccini

**Affiliations:** 1 Graduate Program in Genetics and Molecular Biology, Federal University of Rio Grande do Sul, Porto Alegre, Rio Grande do Sul Brazil; 2 IMASP - Instituto Nacional de Ciência e Tecnologia em Multiômica Aplicada à Saúde de Precisão, Porto Alegre, Rio Grande do Sul Brasil; 3 Medical Genetics Service, Hospital de Clínicas de Porto Alegre, Porto Alegre, Brazil; 4 Laboratory of Genomic Medicine, Experimental Research Center, Hospital de Clínicas de Porto Alegre, Porto Alegre, Brazil; 5 Dermatology Service, Irmandade Santa Casa de Misericórdia de Porto Alegre, Porto Alegre, Brazil; 6 Graduate Program in Medical Sciences, Federal University of Rio Grande do Sul, Porto Alegre, Rio Grande do Sul Brazil; 7 Ophthalmology Service, Hospital de Clínicas de Porto Alegre, Porto Alegre, Brazil; 8 Department of Genetics, Federal University of Rio Grande do Sul, Porto Alegre, Rio Grande do Sul Brazil

**Keywords:** Genetic isolates, Founder effect, Albinism, Amerindian

## Abstract

**Background:**

Oculocutaneous albinism (OCA) is a genetic condition with an estimated global prevalence of 1 in 17,000, though this varies across populations. The Kaingang are a Brazilian Indigenous group belonging to the Macro-Jê language family.

**Objective:**

Report a novel variant *TYR*:c.704A>G; p.*Tyr*235Cys in homozygosity among individuals of Kaingang ethnicity in the state of Rio Grande do Sul, Brazil.

**Methods:**

A commercial next-generation sequencing (NGS-based) panel for hypopigmentation and oculocutaneous albinism was applied to one saliva sample; exome sequencing (ES) was performed using NGS in another individual; and the remaining 14 individuals were genotyped by quantitative real-time (qPCR).

**Results:**

The detection of this variant in 14 individuals from the same Indigenous Territory, within an estimated population of 6,000, yields an allele frequency of 0.05, suggesting a founder effect in this population.

**Discussion/Conclusion:**

The affected individuals were not closely related, suggesting that the recurrence of the variant is not due to recent consanguinity but rather to population structure and inheritance from a common ancestor. In addition, NGS and ES have proven essential for accurate diagnosis of oculocutaneous albinism and for molecular reclassification of cases previously diagnosed clinically. This study contributes to understanding the molecular profile of albinism in Brazilian Indigenous populations, sheds light on historically underrepresented communities in genetic research and has the potential to inform targeted interventions and public health policies for individuals with albinism in these communities.

**Supplementary Information:**

The online version contains supplementary material available at https://doi.org/10.1007/s12687-026-00948-x.

## Introduction

Albinism is a group of rare diseases characterized by variable hypopigmentation and poor vision. Oculocutaneous albinism (OCA) is a genetic condition whose global prevalence varies considerably across populations. The mean prevalence in four African countries was 1 in 4264 (range, 1 in 1755 to 1 in 7900), and in three European countries, the mean was 1 in 12,000 (range, 1 in 10,000 to 1 in 15,000) (Kromberg et al. 2023). Pathogenic variants in at least 21 genes have been identified as causing the 22 distinct forms of albinism described in the literature (Fernández et al. 2021; Garrido et al. 2021). Of these, seven genes are associated with oculocutaneous albinism (OCA), one with ocular albinism (OA), eleven with Hermansky–Pudlak syndrome (HPS), one with Chediak–Higashi syndrome (CHS), and one with FHONDA (foveal hypoplasia, optic nerve decussation defect, and anterior segment dysgenesis syndrome) (Diallo et al. 2025; Lasseaux et al. 2018). Despite its well-known status, there remains a lack of molecular and epidemiological data, particularly in low- and middle-income countries such as Brazil (e.g., Moura et al. 2023). Several studies have described OCA in American Indigenous and African communities (e.g., Kromberg and Kerr 2022; Moura et al. 2023).

OCA1 is caused by pathogenic variants in the Tyrosinase gene (*TYR)* and is the most common form of albinism among white people worldwide (Ma et al. 2023). *TYR* encodes tyrosinase, an enzyme that catalyzes the conversion of tyrosine to DOPA and DOPAquinone, the initial steps in melanogenesis (Körner and Pawelek 1982). The melanosome is a specialized organelle within the melanocyte, the cell that produces the pigment melanin. Melanosome biogenesis and maturation proceed through four progressive stages (Diallo et al. 2025). In stage I, the melanosome begins to form as an early endosomal structure. During stage II, intraluminal fibrils derived from the PMEL protein are formed, providing a scaffold for subsequent melanin deposition. In stage III, melanin synthesis begins as melanogenic components are delivered to the developing melanosome. Stage IV represents the fully melanized and mature melanosome. Thus, stages I to IV reflect the progressive maturation and pigmentation of the melanosome. Stages III and IV are characterized by the activity of the main melanogenic enzymes, *TYR*, *TYRP1*, and *TYRP2/DCT*, which enable melanin synthesis.

Pathogenic variants in TYR impair tyrosinase activity, leading to a buildup of melanin precursors and severely reduced or absent melanin synthesis (Liu et al. 2021). The clinical phenotype of OCA1 is typically characterized by hypopigmented skin and hair, pale irises, and variable degrees of visual dysfunction. Both complete loss-of-function pathogenic variants and hypomorphic variants have been described in the TYR gene (Bjeloš et al. 2024; Kaul et al. 2025). Based on residual enzyme activity and melanin production, OCA1 cases can be subdivided into OCA1A and OCA1B. Patients with the OCA1A subtype lack functional TYR enzymes and exhibit severely reduced pigmentation (which can lead to achromia), whereas patients with the OCA1B subtype carry hypomorphic variants in the TYR gene, resulting in reduced enzymatic activity and variable pigmentation (Marçon and Maia 2019; Ma et al. 2023; Thomas et al. 2023).

The Kaingang are a Brazilian Indigenous group within the macro-Jê linguistic family. Jê speakers are distributed from eastern Amazonia to Brazil’s Central Plateau to the southern plateaus, and they show high genetic similarity (Castro E Silva et al. 2022). The Kaingang live across the states of São Paulo, Paraná, Santa Catarina, and Rio Grande do Sul, residing on more than 30 Indigenous territories (Terras Indígenas, or TI), which represent only a small portion of their traditional lands. Their population was estimated at about 45,000 in 2020 (Quintero and Maréchal 2020). Previous genetic studies have shown that, despite sharing a common Native American ancestry with neighboring Indigenous groups such as the Guarani, the Kaingang exhibit a distinctive genetic profile. Kaingang communities show low genetic differentiation (Coefficient of Genetic Differentiation - GST = 4%), suggesting a relatively recent common ancestry and genetic homogeneity (Marrero et al. 2007).

Molecular tests, although essential for accurate diagnosis, remain costly, making them inaccessible to most social groups, including the indigenous population. These tests are especially relevant because clinical and molecular diagnostic results can differ. Moreover, knowledge of genetic variability among Latin American indigenous populations remains limited.

We report 16 individuals with oculocutaneous albinism from seven families across three Kaingang communities in Southern Brazil. Of these, 15 underwent genetic analysis and were found to carry the same variant in the *TYR* gene, suggesting a founder effect. One individual could not be genetically analyzed because he was absent during sample collection.

## Subjects and methods

### Clinical data

This study assessed 16 individuals (6 adults and 10 children) from three indigenous territories in the state of Rio Grande do Sul, Brazil. Initially, two unrelated individuals from the Kaingang community were referred to the Medical Genetics Service at the Hospital de Clinicas de Porto Alegre (HCPA) for diagnosis and counseling. During pedigree data collection, we identified additional individuals with albinism in the original community. Therefore, in the second phase of the study, a community-based approach was implemented within the indigenous territory. All individuals underwent physical evaluation by medical geneticists and dermatologists. Ophthalmological evaluation was performed using the Topcon TRC-50DX Fundus Camera from IMAGEnet^®^. During this stage, family history and socioeconomic data were collected.

### Genetic analysis

A commercial genetic panel from Invitae/Labcorp based on Next-Generation Sequencing (NGS), including 47 genes related to hypopigmentation disorders and oculocutaneous albinism (Supplementary Table 1), was performed using a saliva sample in one case, and standard Exome sequencing (ES) was carried out by a commercial laboratory in another individual. Exome sequencing was performed using Illumina DNA Prep with enrichment and a customized Twist Biosciences v3 capture kit, followed by sequencing on the Illumina NovaSeq 6000 platform. Reads were aligned to the GRCh38 reference genome using BWA-MEM, and variants were identified using DeepVariant and ExomeDepth. Variant interpretation followed ACMG guidelines and included annotation with the GnomAD population databases. Sequencing quality metrics showed 99.0% of target bases covered by at least 10 reads, a mean coverage depth of 71×, and a total of 24,506,656 reads.

For the remaining 14 individuals, genotyping was performed using real-time PCR. Genotyping of the *TYR* c.704 A > G (rs2496530428) variant was performed using a TaqMan SNP Genotyping Assay (Applied Biosystems, Foster City, CA, USA). Real-time PCR reactions were carried out in a final volume of 12 µL containing TaqMan Genotyping Master Mix, the specific TaqMan SNP Genotyping Assay, and genomic DNA, according to the manufacturer’s instructions. Amplification and allelic discrimination were performed on a StepOnePlus™ Real-Time PCR System (Applied Biosystems, Foster City, CA, USA) under the following cycling conditions: an initial denaturation step at 95 °C for 10 min, followed by 50 cycles of denaturation at 95 °C for 15 s and annealing/extension at 60 °C for 1 min. Genotypes were assigned automatically based on fluorescence signals from allele-specific probes using the StepOne™ Software.

### Hardy–weinberg equilibrium

Given that albinism is a genetic condition with an autosomal recessive inheritance pattern and that the population is in Hardy–Weinberg equilibrium (Hardy 1908), the frequency of the recessive allele (q) was estimated from the prevalence of albinism (q²) using Eq. 1.

$$q=\sqrt{q^2}$$

The estimated frequency of the non-recessive allele was calculated as *p* = 1 - q, and the expected frequency of heterozygotes (2pq) was estimated using Eq. 2.

$$2pq=2\left(1-q\right)q$$

### Compliance with ethics guidelines

All procedures followed were in accordance with the ethical standards of the responsible committee on human experimentation (institutional and national) and with the Helsinki Declaration of 1975, as revised in 2000. We obtained informed consent from all patients to be included in the study. Additional informed consent was obtained from all patients for whom identifying information is included in this article.

This study was approved by the Research Ethics Committee of the Hospital de Clinicas de Porto Alegre (HCPA) and the National Council of Ethics in Research (CONEP) (Protocol number: CAAE 81756224.5.0000.5327). Parents of participants provided written informed consent for photographs, genetic testing, and publication.

## Results

###  Clinical report - individual 1

**Fig. 1:**
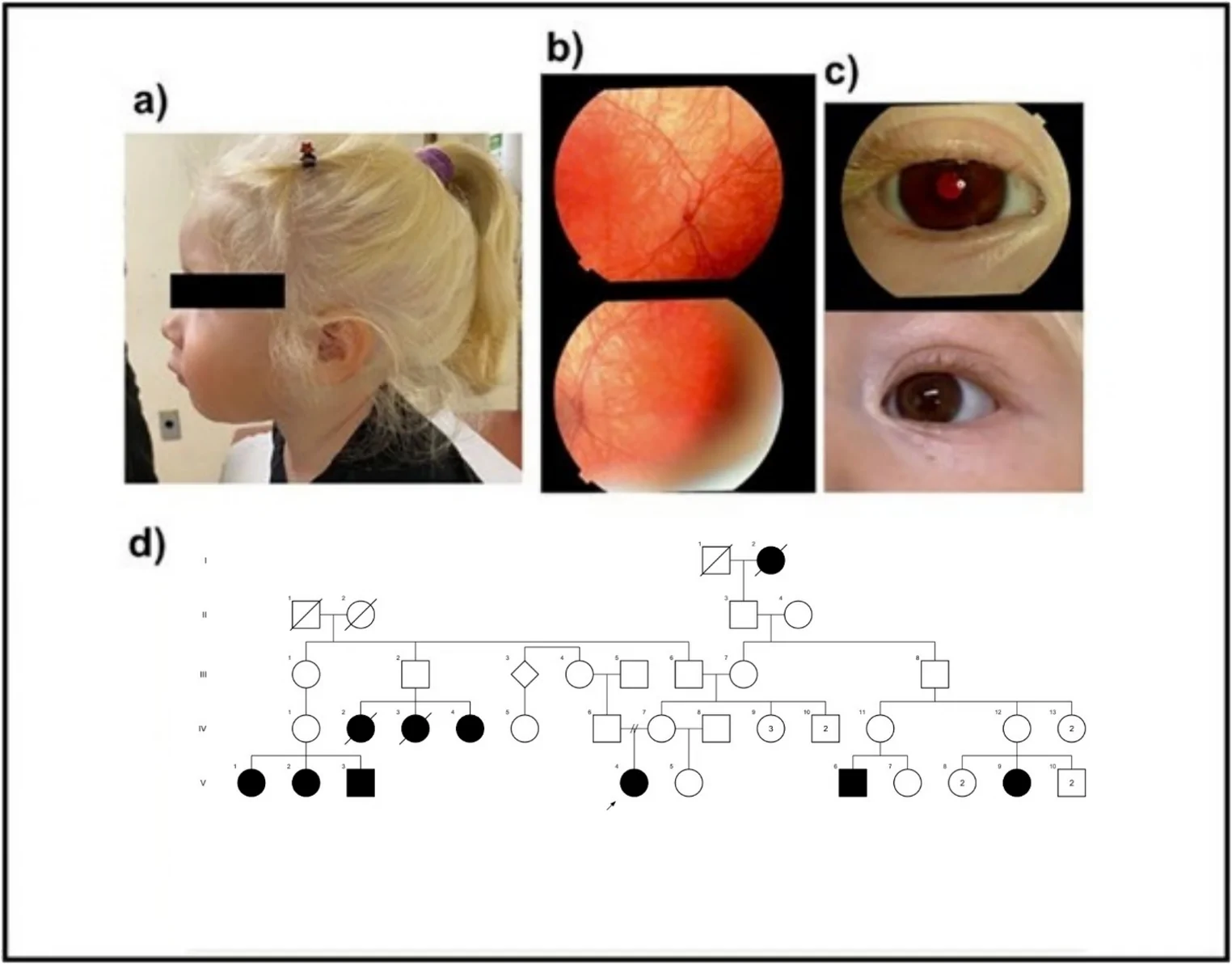
Clinical characteristics from individual 1. (**a**) Details of skin and hair hypopigmentation, (**b**) Details of foveal hypoplasia and fundus hypopigmentation, (**c**) Aspects of iris transillumination defect and Iris with pigmentation. (**d**) Individual’s 1 pedigree with recurrence of albinism in the maternal side. The mother attests that there is no consanguinity among the child’s parents

A 4-year-old girl (first seen at 2 years and 7 months) born on indigenous land to Kaingang parents was referred to the Hospital de Clínicas de Porto Alegre (Porto Alegre, Rio Grande do Sul, Brazil) for evaluation after a clinical diagnosis of OCA2. During her first appointment, the following characteristics were noted: generalized white skin (Fitzpatrick Skin Type 1; Coleman et al. 2023) and brownish irises (Fig. 1a). The ophthalmological evaluation reported pendular nystagmus, divergent strabismus, iris transillumination defect, foveal hypoplasia, and fundus hypopigmentation, which are characteristic of bilateral ocular albinism (Fig. 1b-c). Uncorrected visual acuity was 20/200 in each eye, and binocular visual acuity was also 20/200. Her mother didn’t report consanguinity with the girl’s father but referred to other cases of albinism in her family (Fig. 1d). We couldn’t collect information from the father’s family. Although the phenotypic diagnosis was OCA2 (clinical diagnosis provided by the dermatology team), genetic sequencing identified the *TYR*:c.704 A > G (p.Tyr235Cys) [ClinVar: 2011097] in homozygosity, classified by the commercial lab as a likely pathogenic variant consistent with oculocutaneous albinism type 1 (OCA1). This variant was not previously reported in the literature. Therefore, the commercial laboratory submitted this variant to the ClinVar database and classified it as likely pathogenic (Variation ID: 2011097).

###  Clinical report - individual 2

**Fig. 2:**
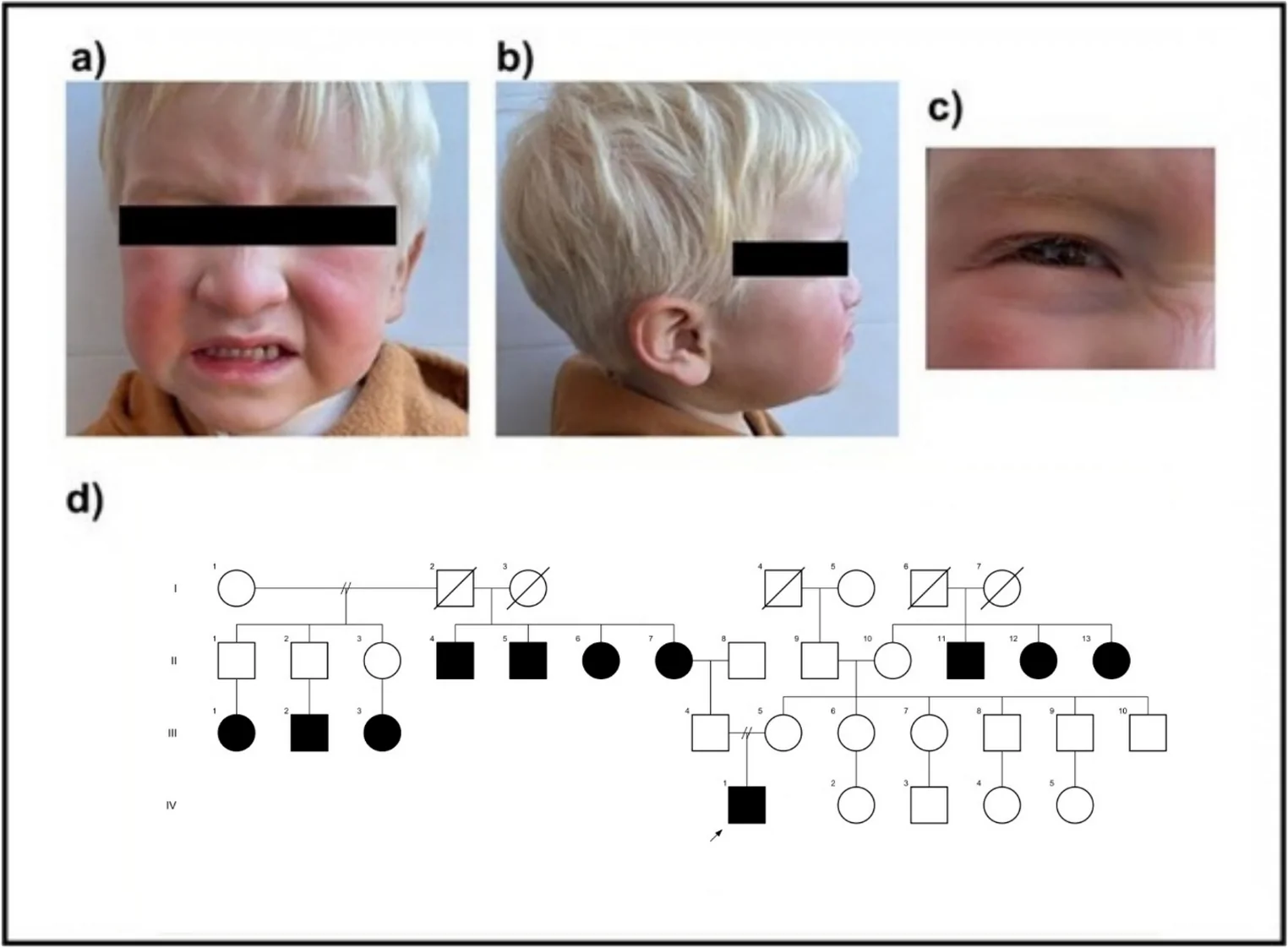
Clinical characteristics from individual 2. (**a**) Detail of skin hypopigmentation. (**b**) Detail of hair hypopigmentation. (**c**) Aspects of iris with pigmentation. (**d**) Individual’s 2 pedigree with recurrence of albinism in maternal side

A two-year-old Kaingang boy (first seen at eight months) was referred to the Hospital de Clinicas de Porto Alegre after a pediatrician diagnosed OCA. His clinical features include generalized white skin and hair and grayish-pink irises. Ophthalmological examinations reported horizontal nystagmus, iris transillumination, a hypopigmented optic disc, and diffuse retinal hypopigmentation (Fig. 2a-c). Visual acuity could not be formally quantified and was assessed qualitatively by visual tracking of light stimuli. His mother and father came from different Kaingang communities (196 km apart). His pedigree is shown in Fig. 2d. Although the parents denied consanguinity, other cases of albinism were present on both the maternal and paternal sides. They didn’t acknowledge any degree of kinship with any of the family members of Individual 1 and live on a different indigenous land, 119 km from the Individual 1 family. Standard exome sequencing (ES) was performed, and the same variant reported in Individual 1 was detected in homozygosity in the *TYR*: c.704 A > G; p.Tyr235Cys.

###  Community approach

Following these two cases, we were informed of additional cases of albinism in a Kaingang community on the Guarita Indigenous Territory (TI Guarita), which has an estimated population of approximately 6,000 individuals (Terras indígenas no Brasil 2026). Given TI Guarita’s extensive size (23,406 hectares), a community-based approach was adopted, with fieldwork conducted by a multidisciplinary team that included community members (medical science students and one graduate student in education). The first visit focused on site identification and community engagement, presenting the project’s objectives and procedures. During a second visit, pedigree and clinical evaluations were conducted for families with individuals affected by albinism, and biological samples were collected via buccal swabs for genotyping. Clinically, all affected individuals exhibited similar features, including cutaneous and hair hypopigmentation, nystagmus, and ocular hypopigmentation, consistent with the findings described in the two initial cases. However, unlike classic OCA1 types, individuals presented with cutaneous nevi containing eumelanin, and some even exhibited sun tanning. Upon dermatological evaluation, 4 of the 14 patients had a history of skin cancer and/or a current diagnosis of skin cancer, one of the main diseases associated with albinism.

**Fig. 3:**
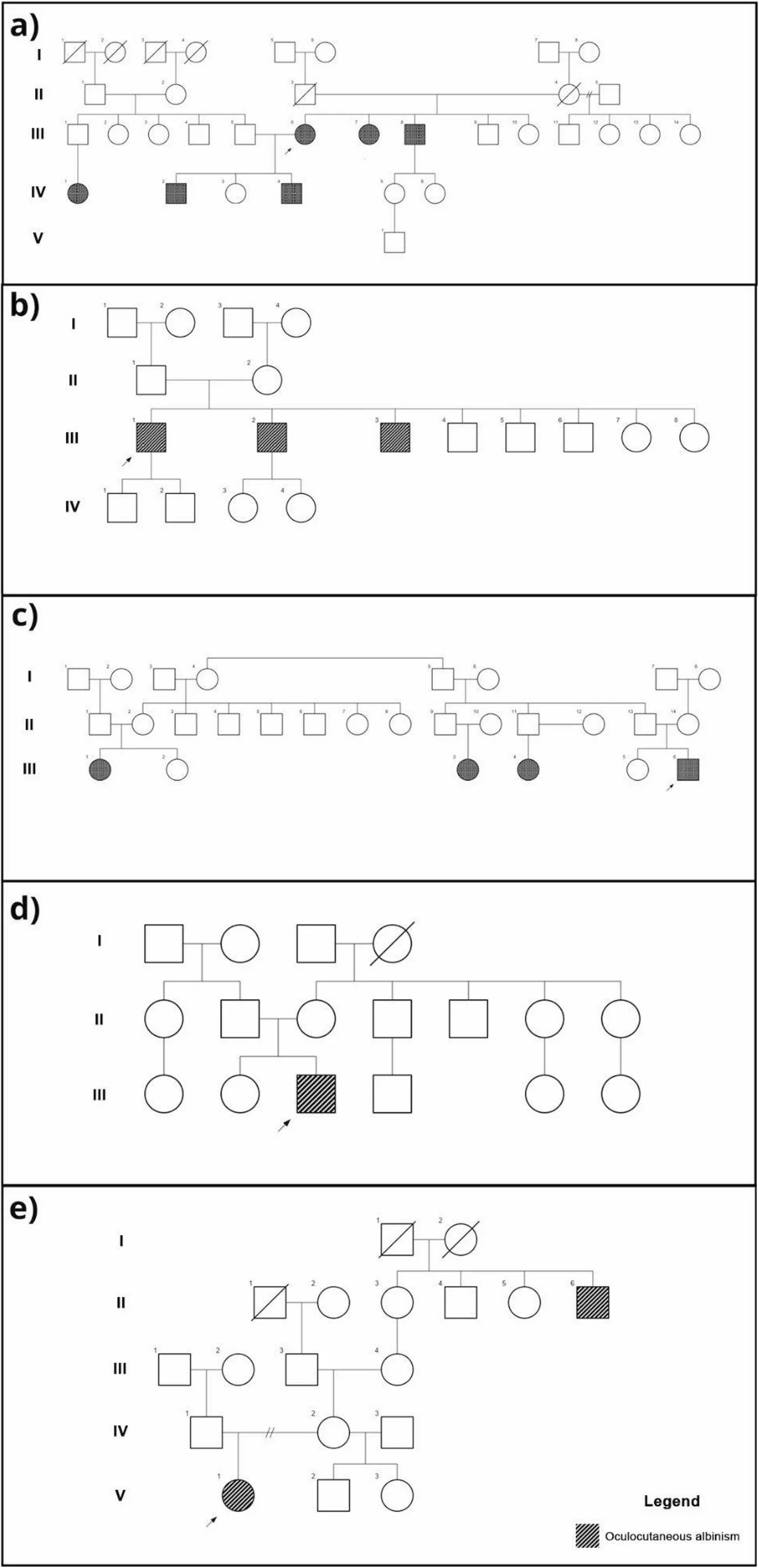
Pedigree of the TI Guarita. **a**, **b**, **c**, **d** and **e** are the studied families. The black arrows indicate the families’ probands

The molecular investigation, based on the founder-effect assumption, aimed to detect the variant using real-time PCR. From the pedigrees, we identified 14 individuals across five distinct families (Fig. 3). The same variant was detected in homozygosity in all individuals with albinism tested.

With an estimated population of 6,000 individuals, the prevalence of albinism in the TI Guarita was approximately 0.23% (1:435). Under the estimated Hardy-Weinberg equilibrium, the allele frequency of the *TYR* variant was estimated at 0.05, with a heterozygote frequency of 0.09 (9%).

**Fig. 4:**
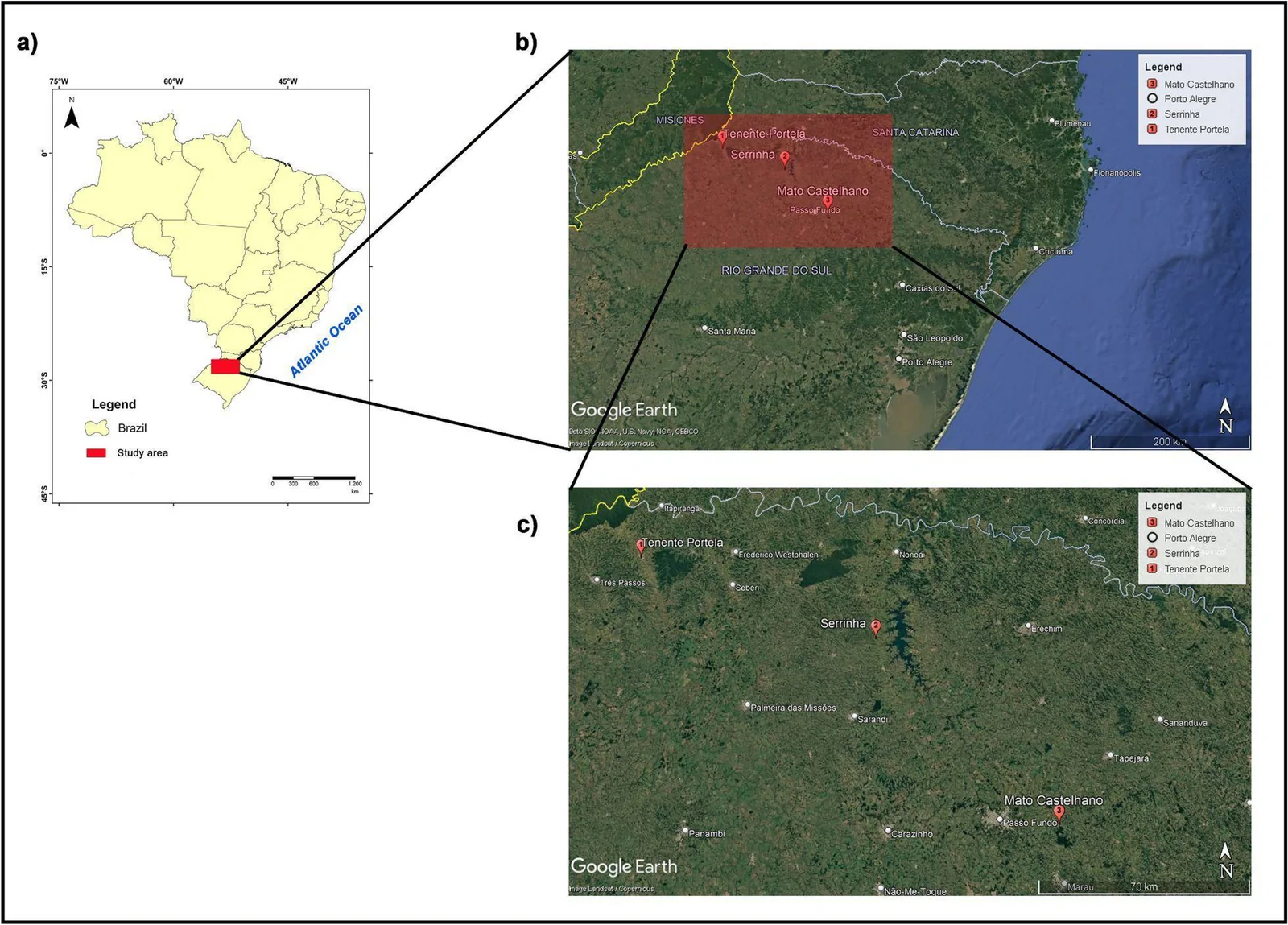
(**a**) Map of Brazil highlighting the studied region. (**b**) Spatial overview of the studied region, emphasizing the distances between the Indigenous Lands. (**c**) Spatial distribution of the Indigenous Lands included in this study, showing communities with reported cases of albinism in Southern Brazil. Source: the authors

The geographic distribution of the Indigenous communities included in this study is shown in Fig. 4A and B, whereas the spatial relationships among these communities, along with their distances from urban centers and healthcare facilities, are shown in Fig. 4C.

###  In silico prediction of variant pathogenicity

 All 16 individuals with albinism analyzed were homozygous for *TYR*:c.704 A > G (p.Tyr235Cys). This variant is absent from population databases, including GnomAD v3 and v4, the Online Archive of Brazilian Mutations (ABraOM), and the Regeneron Genetic Center. The c.704 A > G variant is a missense point mutation in *TYR*, resulting in substitution of an aromatic amino acid (tyrosine) with a polar, reactive, thiol-containing amino acid (cysteine) at position 235 of the tyrosinase protein (*p.Tyr235Cys*). This variant is located in exon 1, within the intramelanosomal domain, specifically in the tyrosinase-like subdomain. The affected residue is conserved across multiple species, underscoring its functional significance. Following the American College of Medical Genetics and Genomics (ACMG) variant classification criteria (Richards et al. 2015; Table 1), in silico predictive analyses suggested a deleterious effect of the variant, yielding a Combined Annotation Dependent Depletion (CADD) score of 26.2 and a REVEL score of 0.89.

**Table 1:** Evidence-based criteria used according to ACMG guidelines for the classification of the *TYR* c.704 A > G variant

Criterion	Justification	Strength	Points
PP3	Multiple in silico tools predict a deleterious effect (REVEL score = 0.89)	Moderate	2
PM5	Different amino acid substitutions at the same residue as a known pathogenic variant	Moderate	2
PM2	Extremely low frequency or absent in population databases (e.g., gnomAD)	Supporting	1
PP4	Phenotype is highly specific for a disease with a single genetic etiology	Supporting	1
PM3	Homozygous occurrence in affected individuals is consistent with autosomal recessive inheritance	Supporting	1
Total			7
Classification	likely pathogenic based on cumulative criteria	—	—

## Discussion

Genetic studies of Kaingang indigenous communities in Rio Grande do Sul, including those on Mendelian conditions, are not unprecedented. In 1958, Francisco Mauro Salzano was already conducting expeditions to villages in northern Rio Grande do Sul, including the Guarita Indigenous Territory (Salzano 1961a). At that time, the indigenous population in the TI Guarita numbered fewer than 1,000, with 14% of unions consanguineous, compared with an estimated 2.4% for the state of RS at the same period. More interestingly, Salzano (1961b) observed a high prevalence of albinism among individuals in Kaingang communities neighboring the Guarita (Cacique Doble and Nonoai). Our community work involved only the TI Guarita, but the two initial cases originated from other villages. A review conducted by our group (Moura et al. 2023) identified additional reports of albinism among indigenous populations in Brazil in the North (Acre), Center West (Mato Grosso), Southeast (São Paulo), and South (Paraná, Rio Grande do Sul). These reports pertain to different ethnic groups, such as Guarani, Mbya-Guarani, Kaxinawa, Kuikuro, and Kaingang; however, no molecular analyses were performed.

In the present study, molecular analysis showed that all 16 individuals tested were homozygous for the same variant in the *TYR* gene. This was expected, given the community’s relative isolation and endogamy. At this time, we cannot infer the ancestral origin of this variant without additional molecular studies. Although isolated, 23% of individuals living in the TI Guarita were classified as admixed in 1958 (Salzano 1961a), and the Kaingang communities have been in contact with individuals of European and African descent since the 19th century (Marrero et al. 2007). Therefore, a gene flow event from a non-indigenous group cannot be ruled out.

Consistent with the high inbreeding, other recessive disorders have also been identified among the Kaingang communities, including galactosemia. Galactosemia is an autosomal recessive disorder caused by variants in the *GALT* gene. and a novel haplotype c.[90dup; 529 A > G] (p.[His31Alafs*9;Met177Val]) was detected only in Kaingang patients. The minimal prevalence of CG in this Indigenous population was estimated in at least 1:900 live births. (Simão-Medeiros et al. 2025).

Although the variant *TYR*:c.704 A > G (p.Tyr235Cys) has not been previously documented, a different pathogenic variant affecting the same amino acid residue has been reported in the literature (Wei et al. 2010; Zhong et al. 2019; Chuan et al. 2021), suggesting potential clinical relevance. In fact, Exon 1 harbors a high number of previously identified pathogenic variants (Wei et al. 2010). Although this novel variant reported here suggests a pathological effect consistent with oculocutaneous albinism type 1 (OCA1), it does not meet all criteria for a pathogenic variant and is still classified as likely pathogenic according to the ACMG variant classification criteria (Richards et al. 2015).

Other indigenous or isolated groups in the Americas have also been reported to have increased prevalence of albinism. In South America, molecular investigations have been conducted in different populations with albinism from Colombia and Brazil. The Colombian study sought to determine the ancestral origins of *TYR* and *OCA2* mutations in patients with OCA from two Andean communities (Cárdenas et al. 2024). These authors found that mutations in *TYR* (seven variants) and *OCA2* (one variant) underscore the genetic complexity of admixed American populations, and that both consanguinity and admixture can contribute to the risk of autosomal recessive OCA in Colombia.

In North America, the Hopi communities in Arizona and New Mexico (USA), the prevalence of albinism is approximately 1:227 (0.44%), one of the highest reported worldwide (Woolf and Grant 1962). In Hopi communities, the high frequency of individuals with albinism was hypothesized to have been introduced by gene flow and maintained within the population by cultural selection (Woolf and Dukepoo 1969).

In Kaingang communities, we could obtain only oral testimonies from local residents indicating that people with albinism were considered “moon warriors,” believed to have special abilities to fight at night. This is not sufficient evidence of positive cultural selection, but it at least indicates that there is no prejudice against people with albinism, as reported in other communities (Hall 2022; Panara 2023).

## Conclusions

The high allelic frequency (0.05) and the high estimated carrier frequency (0.09) indicate a substantial impact of oculocutaneous albinism in this Kaingang community. In addition to tailored medical follow-up, genetic counseling is needed. This process should be pursued through a careful approach that considers the community’s cultural beliefs and unique characteristics.

## Supplementary Information

Below is the link to the electronic supplementary material.

Supplementary Material 1 (DOCX 20.6 KB)

## Data Availability

No datasets were generated or analysed during the current study.
