# Supplementary material for: A novel TYR variant implicated in oculocutaneous albinism type 1 (OCA1): a study in an indigenous population in Southern Brazil
Source: J Community Genet. 2026 Sep 24;17(5):111. doi: 10.1007/s12687-026-00948-x (PMC13612799; doi:10.1007/s12687-026-00948-x)
Supplement: Supplementary file 1 [file 12687_2026_948_MOESM1_ESM.docx]

**Supplementary Materials**

**Supplementary Table 1 –** List of the 47 genes included in the commercial genetic panel from Invitae/Labcorp based on Next-Generation Sequencing (NGS), related to hypopigmentation disorders and oculocutaneous albinism (by Gene/Transcript).

| Gene | Transcript |
| --- | --- |
| *ACD* | NM_001082486.1 |
| *AP3B1* | NM_003664.4 |
| *AP3D1* | NM_001261826.1 |
| *BLOC1S3* | NM_212550.4 |
| *BLOC1S6* | NM_012388.3 |
| *C10orf11* | NM_032024.4 |
| *CLCN7* | NM_001287.5 |
| *CTC1* | NM_025099.5 |
| *DKC1* | NM_001363.4 |
| *DTNBP1* | NM_032122.4 |
| *EDN3* | NM_207034.2 |
| *EDNRB* | NM_000115.3 |
| *EPG5* | NM_020964.2 |
| *FRMD7* | NM_194277.2 |
| *GNAI3* | NM_006496.3 |
| *GPR143* | NM_000273.2 |
| *HPS1* | NM_000195.4 |
| *HPS3* | NM_032383.4 |
| *HPS4* | NM_022081.5 |
| *HPS5* | NM_181507.1 |
| *HPS6* | NM_024747.5 |
| *KIT* | NM_000222.2 |
| *KITLG* | NM_000899.4 |
| *LYST* | NM_000081.3 |
| *MITF* | NM_000248.3 |
| *MLPH* | NM_024101.6 |
| *MYO5A* | NM_000259.3 |
| *NHP2* | NM_017838.3 |
| *NOP10* | NM_018648.3 |
| *OCA2* | NM_000275.2 |
| *PARN* | NM_002582.3 |
| *PAX3* | NM_181457.3 |
| *RAB27A* | NM_004580.4 |
| *RET* | NM_020975.4 |
| *RTEL1* | NM_001283009.1 |
| *SLC24A5* | NM_205850.2 |
| *SLC38A8* | NM_001080442.2 |
| *SLC45A2* | NM_016180.4 |
| *SNAI2* | NM_003068.4 |
| *SOX10* | NM_006941.3 |
| *TERC* | NR_001566.1 |
| *TERT* | NM_198253.2 |
| *TINF2* | NM_001099274.1 |
| *TYR* | NM_000372.4 |
| *TYRP1* | NM_000550.2 |
| *USB1* | NM_024598.3 |
| *WRAP53* | NM_018081.2 |

**Supplementary Table 2 –** Phenotype and genotype of all evaluated individuals.

| Individual ID | Community/Region | Sex | Age | Family history of albinism | Phenotype (exact clinical description) | Analytical method | Skin lesions/injury | History of skin cancer | Variant(s) identified | Ophthalmologic findings |
| --- | --- | --- | --- | --- | --- | --- | --- | --- | --- | --- |
| K01 | Serrinha | F | 06 | Yes |  | Targeted albinism gene panel | None | No | *TYR*:c.704A>G; p.Tyr235Cys |  |
| K02 | Mato Castelhano | M | 03 | Yes |  | Exome sequencing | None | No | *TYR*:c.704A>G; p.Tyr235Cys |  |
| KTP01 | Tenente Portela/Pau escrito | M | 35 | Yes | White skin with no tanning ability; white hair with a yellowish tint | qPCR | Not reported | Yes, on the back in 2015 | *TYR*:c.704A>G; p.Tyr235Cys | Green irises; nystagmus present; no strabismus |
| KTP02 | Tenente Portela/Pau escrito | M | 33 | Not reported | Not reported | Not available | Not reported | Not reported | Not available | Not available |
| KTP03 | Tenente Portela/Pau escrito | M | 34 | Yes | White skin with no tanning ability; white hair with a yellowish tint | qPCR | Sunburns | No | *TYR*:c.704A>G; p.Tyr235Cys | Brown-green irises; nystagmus present; no strabismus |
| KTP04 | Tenente Portela/Pau escrito | M | 40 | Yes | White skin with no tanning ability; white hair with a yellowish tint | qPCR | Yes – neck inflammation | Yes | *TYR*:c.704A>G; p.Tyr235Cys | Green irises; nystagmus and strabismus present |
| KTP05 | Tenente Portela/Pau escrito | F | 35 | Yes | White skin with no tanning ability; white hair with a yellowish tint | qPCR | None | No | *TYR*:c.704A>G; p.Tyr235Cys | Green irises; nystagmus and strabismus present |
| KTP06 | Tenente Portela/Pau escrito | F | 39 | Yes | White skin with no tanning ability; white hair with a yellowish tint | qPCR | Sunburns | Yes | *TYR*:c.704A>G; p.Tyr235Cys | Green irises; nystagmus present; no strabismus |
| KTP07 | Tenente Portela/Pau escrito | M | 19 | Yes | White skin with no tanning ability; white hair with a yellowish tint | qPCR | Skin wounds | Yes | *TYR*:c.704A>G; p.Tyr235Cys | Brown-green irises; nystagmus present; no strabismus |
| KTP08 | Tenente Portela/Pau escrito | M | 12 | Yes | White skin with no tanning ability; white hair with a yellowish tint | qPCR | None | No | *TYR*:c.704A>G; p.Tyr235Cys | Not reported |
| KTP09 | Tenente Portela/Estiva | M | 05 | No | White skin with no tanning ability; white hair with a yellowish tint | qPCR | Skin wounds at age 2 | No | *TYR*:c.704A>G; p.Tyr235Cys | Brown irises; mild nystagmus present; no strabismus |
| KTP10 | Tenente Portela/KM 10 | F | 09 | Yes | White skin with no tanning ability; white hair with a yellowish tint | qPCR | Sunburns | Not reported | *TYR*:c.704A>G; p.Tyr235Cys | Gray-green irises; nystagmus and strabismus present |
| KTP11 | Tenente Portela/Linha Esperança | F | 04 | Yes | White skin with no tanning ability; white hair with a yellowish tint | qPCR | None | No | *TYR*:c.704A>G; p.Tyr235Cys | Brown-green irises; nystagmus present; no strabismus |
| KTP12 | Tenente Portela/Linha Esperança | M | Not reported | Not reported | White skin with no tanning ability; white hair with a yellowish tint | qPCR | Not reported | Not reported | *TYR*:c.704A>G; p.Tyr235Cys | Not reported |
| KTP13 | Tenente Portela/Linha Esperança | F | 07 | Yes | White skin with no tanning ability; white hair with a yellowish tint | qPCR | Sunburns | No | *TYR*:c.704A>G; p.Tyr235Cys | Brown-green irises; nystagmus present; no strabismus |
| KTP14 | Tenente Portela/Linha Esperança | F | 04 | Yes | White skin with no tanning ability; white hair with a yellowish tint | qPCR | None | No | *TYR*:c.704A>G; p.Tyr235Cys | Light brown irises; nystagmus present; no strabismus |

Abbreviations: qPCR, quantitative polymerase chain reaction; F, female; M, male; *TYR*, tyrosinase. Variant nomenclature: c., coding DNA sequence; p., protein-level change.
